# Microelectrodes for Atrial Flutter: Bringing a Scalpel to a Gun Fight

**DOI:** 10.19102/icrm.2018.090607

**Published:** 2018-06-15

**Authors:** Rahul N. Doshi

**Affiliations:** ^1^Division of Cardiovascular Medicine, Keck School of Medicine of USC, Los Angeles, CA, USA

**Keywords:** Ablation, atrial flutter, large-tip electrodes, microelectrodes

In the current issue of *The Journal of Innovations in Cardiac Rhythm Management*, Somani et al. present a comparison between using information generated from microelectrode recordings on an 8-mm-tip ablation catheter and using standard bipolar electrode recordings for their maximum-voltage technique for the ablation of atrial flutter (AFL).^1^

To appreciate the findings of this manuscript, one must first understand the approach. In 2006, Redfearn et al. described the technique, which is based on the anatomic findings that the cavotricuspid isthmus (CTI) consists of discreet muscle bundles interspersed with fibrous connective tissue.^2^ Using conventional bipolar recordings from either an 8-mm nonirrigated-tip or a 5-mm externally irrigated-tip ablation catheter, they targeted the sites with maximum voltage to ablate along a traditional “6 o’clock” position. They demonstrated that the use of this technique prompted a change in conduction patterns in the majority of patients with a series of discreet “jumps” in activation time across the CTI until full block was achieved, negating the need for a contiguous ablation line. They hypothesized that this would result in less unnecessary ablation and shorter ablation times as compared with a traditional “drag” technique.

Somani et al. compared this approach using conventional bipolar recordings with the same approach using microelectrodes on the IntellaTip MiFi™ 8-mm-tip catheter (Boston Scientific, Natick, MA, USA).^1^ Microelectrode recordings have the ability to more accurately detect near-field electrograms as compared with traditional bipolar electrodes and have been hypothesized to more accurately identify viable tissue in patients undergoing redo procedures for AFL.^3^ However, data showed that using microelectrode recordings for this technique resulted in longer overall procedure and fluoroscopy times. Further analysis might suggest that these findings were a result of significant differences amongst patients who presented in AFL.

In reading this study, the reader should not think that the MiFi™ catheter (Boston Scientific, Natick, MA, USA) was demonstrated to be inferior to a standard 8-mm catheter for the ablation of AFL. The study’s sample size is small, and not truly statistically powered to show any differences in outcome, especially independent of traditional techniques. Still, the “clue” as to why microelectrodes perhaps should not be used for the maximum voltage approach may lie in the decreased power used for ablation of the study group. The authors hypothesized that the reason for less effectiveness may be that the lesion delivered might have been slightly more proximal than that created when using conventional recordings. While this might be the case, it would not explain decreased power delivery in a temperature-limited mode of ablation. For this, it is worth considering several simple biophysical principles of radiofrequency ablation.

## Reviewing the mechanics

In their seminal paper, Nath et al. established many of the fundamental properties of radiofrequency catheter ablation.^4^ Temperature at the tip–tissue interface is proportional to lesion size, but this effect is dependent on several factors including electrode contact, orientation, tip electrode surface cooling, and the location of temperature measurement. The use of large electrodes and variable contact are known to produce nonuniform lesions, and Haines has demonstrated that increasing the number of thermistors or thermocouples for greater control yields smaller lesions for constant generator settings.^5^ A detailed comparison of ablation technologies and lesion volume/depth for different power settings was performed by Everett et al.^6^ They demonstrated that ablation lesion depth and volume increased with the amount of power delivered and that, with all catheters, complications are more likely to occur with increased temperature at the tip–tissue interface.

The IntellaTip MiFi™ 8-mm-tip catheter (Boston Scientific, Natick, MA, USA) has the thermistor located at the distal tip of the ablation electrode. This location would result in rapid temperature increase and, thus, a limitation in power delivery when the tip is in contact with relatively nonconductive fibrous tissue, such as when the catheter is relatively perpendicular to the tissue plane but in a groove between pectinate muscle bands. However, the microelectrodes located 2 mm from the distal tip could record large discreet near-field signals and, thus, be considered a target for ablation. These recordings, when compared with mapping for the largest voltages on bipolar recordings, could result in ablation occurring in between the muscle bundles and, therefore, a less effective ablation outcome. The effect might be magnified in AFL given the more uniform wavefront and greater conduction velocity across the CTI when compared with sinus rhythm. Itoh et al. demonstrated that electrogram amplitude correlates with conduction velocity in common AFL.^7^

For ablation of the CTI, it is desirable to create lesions of significant depth and volume. It has been demonstrated that large-tip electrodes result in shorter procedure times with equivalent efficacy to irrigated ablation catheters.^8^ Other studies have shown equivalent procedure times and efficacy.^9^ These technologies have shown greater short- and long-term success rates when compared with standard radiofrequency ablation catheters or cryoablation catheters.^10,11^ If one is creating larger lesions with comparable safety and greater efficacy than that seen with conventional ablation,^12^ is the use of “surgical precision” really necessary? Given the relative safety of ablation on the CTI, it might not be.

The addition of microelectrodes to an ablation catheter may allow for more accurate near-field recordings than with the large “antenna” of a bipolar recording, especially with an 8-mm tip. However, this might be analogous to placing a magnifying glass on a sledgehammer. The approach employed by Somani et al. is reminiscent of placing multiple thermistors on a large-tip electrode, yielding greater safety at the cost of smaller lesions. This is analogous to the saying “don’t bring a knife to a gunfight” (or, in this case, a scalpel). Maybe the utility of such technology is in ablation procedures in which the risk of collateral damage is of greater importance, such as in the ablation of atrial fibrillation.
